# Surgery

**Published:** 1909-12

**Authors:** H. G. Kyle


### SURGERY.

**Wounds of the heart.**—It is rarely that the opportunity is presented for personal experience in dealing with wounds of the heart. About 160 cases of operation for this condition have been recorded since Farina, of Rome, published his case in 1896, and Charles H. Peck[2] has recently added another case. The patient was seen about half an hour after the accident. She was profoundly shocked, and no radial pulse could be felt. The area of heart dulness was increased. There was a stab wound at the left border of the sternum, over the third costal cartilage, which bled very little. Operation was commenced within forty-five minutes after the receipt of the injury. A quadrangular flap, with the base external, its margins at the second interspace, left edge of sternum and fifth interspace, was rapidly reflected, the fourth, fifth, and sixth costal cartilages were cut at their sternal attachments. The internal mammary vessels were ligatured above and below. The ribs were cut at their costochondral junction to form a hinge, and the musculo-cartilaginous flap turned outwards, the pleura being separated with a swab.

Intra-pericardial tension was so great, that the heart beat could not be felt. On opening the sac, 300 cc. of dark blood escaped; there was immediate return of the radial pulse. A

[2] *Ann. Surg.*, 1909, l, 100.

wound about one c.m. long was found in the right auricle; a stream of blood spurted from this at each systole. The hemorrhage was controlled after the introduction of four sutures. The rapid tumbling action of the heart rendered the stitching somewhat difficult. The wound was closed without drainage. Intravenous saline injections were given. The patient recovered, and left the hospital twenty-four days after the operation.

Vaughan[1] narrates the case of a man aged thirty-two with a wound half an inch long, just internal to the left nipple, between the fourth and fifth ribs, very little blood escaping. He was semi-comatose, partly from drink. Pulse 120. Heart sounds very feeble. Operation performed a little more than an hour after the infliction of the injury.

A quadrangular flap was raised containing the fourth, fifth, and sixth costal cartilages, the hinge being internal. In raising the flap the pleura was widely opened and the lung collapsed. The wound passed through the lung and into the right ventricle. There was a little blood in the pericardium, and about a pint in the pleural cavity. The wound was closed with a continuous silk suture. Some bleeding occurred from the stitch holes; this was stopped by a second row of Lembert stitching. No drainage was used. The pulse was better after the operation. The patient developed *delirium tremens*, but eventually recovered. The writer tabulates all the recorded cases, noting the important features of each.

Wounds of the ventricle are very much more frequent than wounds of the auricle, in the proportion of 136 to 8. The two ventricles were wounded with equal frequency. All writers agree that wounds of the auricle are more dangerous than those of the ventricle.

The incision selected should be one that gives the most free exposure of the heart, and is least likely to open the pleural cavity. Cases have been dealt with by incisions through an intercostal space, and by resection of portions of costal cartilage and sternum; but these methods are not satisfactory, they do not give free access and entail a much longer operation. The best method is to raise a quadrangular flap, as in the two cases recorded above. The hinge may be external or internal; the latter is to be preferred, as the pleura is more easily reflected without being opened. If more room is required, portions of the sternum can be easily removed after reflection of the flap.

In wounds of the right side, the flap can be commenced to the right of the sternum, a portion of this bone being included.

The internal mammary artery requires ligation.

It is generally impossible to be sure whether the suture penetrates the endocardium or not; it is well to endeavour to avoid doing so, but it does not appear to matter much.

[1] *J. Am. M. Ass.*, 1909, lii, 429.

The tumultuous action of the heart renders the introduction of sutures somewhat difficult; this may be overcome by inserting tension sutures to steady the organ, and by holding it in the hand. It is not possible to insert stitches during diastole only, the action is too rapid, in fact it is rarely possible to say during which part of the cycle they are inserted. In some cases of heart failure massage or stimulation appears to have been useful, in many it has had no apparent effect. Drainage should not be used; hæmostasis should be obtained by suture; drainage is then not only useless, but dangerous as a source of infection. The mortality of operated cases is 65 per cent., mostly from hemorrhage; 20 per cent. were moribund beforehand; others died from sepsis, resulting in pericarditis, pleurisy, and pneumonia. Over 50 per cent. had some infection.

The diagnosis may be difficult or impossible.

The position of the external wound is not a safe guide. If there is doubt, the external wound should be enlarged to ascertain if it has penetrated the chest wall; if this be the case, and there be symptoms of hemorrhage or pressure on the heart, the operation should be proceeded with. If the wound involves the pleura as well as the heart, there will be anæmia and pneumo-thorax. A splashing sound indicates the presence of blood and air in the pericardial sac. In only a comparatively small proportion of cases will surgical intervention be possible. In the larger wounds fatal hemorrhage rapidly takes place. In the smaller wounds the fatal issue results from heart "tamponade," the intra-pericardial pressure preventing entrance of blood through the great veins, with consequent pumping dry of the ventricles.

Travers[1] recorded a case in which a boy lived for ten days after suture of an irregular lacerated wound $2\frac{1}{4}$ in. in extent in the right ventricle. It was caused by his being impaled on the spike of a railing. A portion of the sternum was lodged in the wound and partially controlled the hemorrhage. On removing this there was profuse bleeding, which was checked by insertion of three fingers into the wound. The suture finally stopped the bleeding. It is not quite evident from the account what the actual cause of death was. The *post mortem* revealed a fibrinous exudate in the pericardium, and there had been a free serous discharge from the wound, which suggests sepsis. The record of temperature is not given. One rigor is reported.

H. G. KYLE.

### OBSTETRICS.

**The occurrence of red degeneration in fibromyomata of the uterus during pregnancy.**—Considerable attention has been recently directed to the occurrence of this remarkable change in uterine fibroids. Although the phenomenon is met with in

[1] *Lancet*, 1906, ii, 706.

